# Identification of Functional Interactome of Colistin Resistance Protein MCR-1 in *Escherichia coli*

**DOI:** 10.3389/fmicb.2020.583185

**Published:** 2021-01-25

**Authors:** Hui Li, Yingyu Wang, Qiyan Chen, Xi Xia, Jianzhong Shen, Yang Wang, Bing Shao

**Affiliations:** ^1^Beijing Key Laboratory of Diagnostic and Traceability Technologies for Food Poisoning, Beijing Center for Disease Prevention and Control, Beijing, China; ^2^Beijing Advanced Innovation Center for Food Nutrition and Human Health, College of Veterinary Medicine, China Agricultural University, Beijing, China

**Keywords:** MCR-1, colistin resistance, interactome, co-immunoprecipitation, *E. coli*

## Abstract

The emergence and worldwide dissemination of plasmid-mediated colistin resistance gene *mcr-1* has attracted global attention. The MCR-1 enzyme mediated colistin resistance by catalyzing phosphoethanolamine (PEA) transfer onto bacterial lipid A. However, the interaction partners of MCR-1 located in membrane protein in *E. coli* are unknown. Co-immunoprecipitation (Co-IP) and Mass Spectrometry were performed to define the interacting proteins of MCR-1. A total of three different anti-MCR-1 monoclonal antibody (mAbs) were prepared and 3G4 mAb was selected as the bait protein by compared their suitability for Co-IP. We identified 53, 13, and 14 interacting proteins in *E. coli* BL21 (DE3) (pET28a-*mcr-1*), *E. coli* BL21 (DE3) (pET28a-*mcr-1-200*), and *E. coli* DH5α (pUC19-*mcr-1*), respectively. Six proteins, including the stress response proteins DnaK (chaperone protein) and SspB (stringent starvation protein B), the transcriptional regulation protein H-NS, and ribosomal proteins (RpsE, RpsJ, and RpsP) were identified in all these three strains. These MCR-1-interacting proteins were mainly involved in ribosome and RNA degradation, suggesting that MCR-1 influences the protein biosynthesis through the interaction with ribosomal protein. Multidrug efflux pump AcrA and TolC were important interacting membrane proteins of MCR-1 referred to drug efflux during the PEA modification of the bacterial cell membrane. Overall, we firstly identified the functional interactome profile of MCR-1 in *E. coli* and discovered that two-component AcrA-TolC multidrug efflux pump was involved in *mcr-1*-mediated colistin resistance.

## Introduction

*mcr-1*, as an important plasmid-borne colistin resistant gene, has attracted much attention in recent years for its threat in the clinical efficacy of the last-resort antibiotic when treating multidrug–resistant (MDR) Gram-negative bacterial infections (Liu et al., 2016; Shen et al., 2016; Poirel et al., 2017). *mcr-1* encodes a member of the family of phosphoethanolamine (PEA) transferases that decorates the lipid A headgroups of lipopolysaccharide of the outer membrane of Gram-negative bacteria through the addition of PEA (Anandan et al., 2017; Li et al., 2018). MCR-1 belongs to the alkaline phosphatase superfamily/sulphatase with five transmembrane segments. Many MDR gram-negative bacteria possess multiple members of this family of enzymes that are engaged in the decoration of lipid A or the conserved inner core of the lipopolysaccharide (Needham and Trent, 2013). The proper function of an organism is orchestrated by highly complex protein networks and the interactome is crucial for understanding structural and functional organization in time and space (Sanchez et al., 1999). Deciphering the interactome profile of MCR-1 may help us in decoding its exact physiological function and various related biological process.

At present, the dissemination and prevalence of *mcr-1* has been widely reported in human, foods, animals, and environment (Gao et al., 2016; Ye et al., 2016; Wang et al., 2017). As an important resistant protein, the functional interactome of membrane protein MCR-1 was poorly understood. Affinity purification based on co-immunoprecipitation (Co-IP) coupled to mass spectrometry has become an important method of identifying protein interactome (Pankow et al., 2016; Maccarrone et al., 2017). Discovering interaction protein is still challenging, however, because a comprehensive interactome analysis requires high yields of the bait protein for robust co-purification of its interactors. In this study, we express and purify the catalytic domain and full-length protein of MCR-1, and prepare three different anti-MCR-1 monoclonal antibody (mAbs). We define the interacting proteins of MCR-1 using Co-IP and mass spectrometry in *E. coli* and characterize the protein-protein interaction (PPI) network of MCR-1.

## Materials and Methods

### Expression and Purification of MCR-1

The gene encoding *mcr-1*, was amplified by polymerase chain reaction (PCR) from *E. coli* strain SHP45 genomic DNA using primers pET28a-mcr-1-F and pET28a-mcr-1-R and cloned into the plasmid pET28a to create a high copy expression vector with a N terminal hexa-histidine tag (pET28a-*mcr-1*) (Supplementary Table 1). Then the constructed plasmid pET28a-*mcr-1* was chemically transferred into *E. coli* BL21 (DE3) strains to express the full length MCR-1. For the expression of the catalytic domain of MCR-1, the constructed plasmid pET28a-*mcr-1-200* using primers pET28a-mcr-1-200-F and pET28a-mcr-1-200-R was transformed into *E. coli* BL21 (DE3) strains (Supplementary Table 1). Transformants were selected by the inclusion of kanamycin (50 μg/ml) on Luria Broth (LB) agar plates. The construction of *E. coli* cloning strains [*E. coli* DH5α (pUC19) and DH5α (pUC19-*mcr-1*)] was referred to our previous article (Li et al., 2019). The bacterial strains and plasmids used in this study were listed in Table 1. A starter culture was prepared by inoculating a single colony into 2 ml LB medium and grown at 37°C overnight. A sufficient volume of the starter culture was used to inoculate 200 ml LB medium and grown at 37°C in a shaking incubator set at 200 rpm until the OD_600_ reached 0.5–0.6. Protein expression was induced by the addition of isopropyl β-D-1-thiogalactopyranoside (IPTG) to a final concentration of 0.5 mM and the culture was continued at 18°C, 160 rpm for a further 14 h. The cells were harvested by centrifuging at 9,000 × *g* for 20 min at 4°C.

**TABLE 1:** Bacterial strains and plasmids used in this study.

Plasmids or strains	Description	Source
**General plasmids**		
pET28a	T7 *lac* promotor-operator, N-terminal His tag, kan^r^	Novagen
pUC19	M13 *lac* promoter-operator, amp^r^	Takara
pET28a:His-*mcr-1*	pET28a derivative carrying *mcr-1*	This study
pET28a:His-*mcr-1-200*	pET28a derivative carrying *mcr-1-200* (201–541 bp)	This study
pUC19:*mcr-1*	pUC19 derivative carrying *mcr-1*	Liu et al., 2016
**Strains**		This study
*E.coli*		
DH5α	*endA hsdR17 supE44 thi-1 recA1 gyrA relA1*Δ *(lacZYA-argF)U169 deoR* [Φ*80dlac*Δ *(lacZ)M15*]	Takara
BL21 (DE3)	F^–^ *ompT hsdS_*B*_ (r_*B*_^–^ m_*B*_^–^) gal dcm met* (DE3)	Transgen Biotech
DH5α (pUC19)	*E. coli* DH5α strain carrying plasmid pUC19	Liu et al., 2016
DH5α (pUC19-*mcr-1*)	*E. coli* DH5α strain carrying plasmid pUC19:*mcr-1*	Liu et al., 2016
BL21 (DE3) (pET28a-*mcr-1*)	*E. coli* BL21 (DE3) strain carrying pET28a:*mcr-1*	This study
BL21 (DE3) (pET28a-*mcr-1-200*)	*E. coli* BL21 (DE3) strain carrying pET28a:*mcr-1-200* (201–541 bp)	This study

*^*r*^Indicates resistance.*

The harvested cells were re-suspended in 50 mM sodium phosphate buffer pH8.0 containing 300 mM NaCl, 10 mM imidazole at 4°C. Between 5 and 10 ml was used for each gram of cell pellet and cell lysis was performed using VCX105 ultrasonic instrument (Sonics & Materials, Inc., Newtown, CT, United States). The lysate was centrifuged at 12,000 × *g* for 30 min at 4°C to remove the non-lysed cells. The protein was purified from the supernatant using Ni-NTA agarose beads (Qiagen, Hilden, Germany). Briefly, the supernatant was incubated with pre-equilibrated Ni-NTA agarose beads (1 ml per pellet from a 200 ml culture) for 1 h. The beads were then loaded on a column and washed with 50 mM NaH_2_PO_4_, 300 mM NaCl, and 20 mM imidazole (pH 8.0). Protein was eluted with four column volumes of 50 mM NaH_2_PO_4_, 300 mM NaCl, and 150 mM imidazole (pH8.0). Imidazole was removed from the eluted protein by exchanging buffer to 50 mM NaH_2_PO_4_, 300 mM NaCl, and 1 mM tris (2-carboxyethyl) phosphine (TCEP) (pH8.0) using a PD-10 desalting column (GE Healthcare).

### Preparation of MCR-1 mAb

The purified MCR-1 catalytic protein was used for immunization and custom production of the affinity-purified mouse mAb was used for subsequent experiments. All animal treatments were in accordance with Chinese laws and guidelines that were approved by the Animal Ethics Committee of China Agricultural University. Six eight-week old female BALB/c mice were immunized with the immunogen. The mice were immunized three times with an interval of 3 weeks between immunizations. The collected ascites were further purified to obtain the anti-MCR-1 mAb using AKTA Pure 150 system (GE Healthcare, Buckinghamshire, United Kingdom). Specificity of the antibody was determined by SDS-PAGE and Western blot.

### Co-immunoprecipitation Assay

To identify the interactome profile associated with MCR-1, three *E. coli* clone strains carrying *mcr-1 gene* [DH5α (pUC19-*mcr-1*), BL21 (DE3) (pET28a-*mcr-1*), and BL21 (DE3) (pET28a-*mcr-1-200*)] and two empty vector strains [DH5α (pUC19) and BL21 (DE3) (pET28a)] were included to pull down MCR-1 and associated proteins using Co-IP assays with anti-MCR-1 mAb 3G4 as the bait. Anti-IgG and empty vector pull-downs were set as negative controls. The Co-IP assays of membrane protein MCR-1 was performed as previously described (Pankow et al., 2016). Briefly, the cells of two cloning strains [(DH5α (pUC19), DH5α (pUC19-*mcr-1*)] and three expressing strains [BL21 (DE3) (pET28a), BL21(DE3) (pET28a-*mcr-1*), and BL21(DE3) (pET28a-*mcr-1-200*)] were lysed by 50 mM Tris–HCl (pH7.5), 300 mM NaCl, 1 mM phenylmethylsulfonyl fluoride (PMSF), and 1% *n*-dodecyl β-D-maltoside (DDM). 1 mg of solubilized proteins were incubated overnight with 250 μg of anti-MCR-1 mAb in a reaction volume of 100 μl at 4°C with gentle mixing. 50 μl of pre-equilibrated *G*-Sepharose (GE Healthcare, Uppsala, Sweden) was added and incubated for 4 h at 4°C with gentle agitation. After centrifugation, samples were washed 5 times with solubilization buffer and co-precipitates were eluted by incubation with 0.2 M glycine (pH2.3) and 0.5% Igepal CA-630 (Sigma-Aldrich, St. Louis, MO, United States). Co-precipitates were separated by SDS-PAGE, and visualized with silver staining. The MCR-1 binding proteins were identified using nano LC-MS/MS analysis. Three biological repeats were conducted in the Co-IP assay.

### Nano LC-MS/MS and Data Analysis

The co-precipitates were analyzed on a Q-exactive mass spectrometer equipped with a Dionex Ultimate 3000 Nano (Thermo Fisher Scientific). Peptides were trapped on a Acclaim PePmap 100 (75 μm × 2 cm, nanoviper, C_18_, 3 μm, Thermo Fisher Scientific) and separated on a Venusil XBP C_18_ (2.1 × 150 mm, 5 μm, Agela Technologies) using a gradient formed between solvent A (0.1% formic acid in water) and solvent B (0.1% formic acid in acetonitrile). The gradient started at 5% solvent B and the concentration of solvent B was increased to 80% within 60 min. Database search was performed using the MASCOT software against the *E. coli* K12 database.

### Surface Plasmon Resonance Interaction Analysis of MCR-1 and SspB Protein

The surface plasmon resonance (SPR) interaction analysis was performed using the Reichert 4SPR instrument (Reichert Life Sciences). The MCR-1 and MCR-1-200 protein in PBS buffer were directly immobilized on a Planar Mixed SAM biosensor chip (Reichert Life Sciences), respectively. SspB was sequentially diluted in running buffer PBST (pH7.4, containing 0.05% Tween-20) and injected to be trapped on the chip through the immobilized MCR-1. Evaluation and calculation of the binding parameters were carried out according to the formula: K_*D*_ = K_*a*_/K_*d*_ (K_*a*_ = association rate constant and K_*d*_ = dissociation rate constant).

### Bioinformatics Analysis

A protein-protein interaction (PPI) network was constructed from the STRING database v11.0 (Szklarczyk et al., 2017) using the set of MCR-1 interacting proteins identified here. The interaction score was set as medium confidence (more than 0.400), and gene ontology (GO) enrichment was calculated by Blast2GO software. Metabolic pathway of MCR-1 interacting proteins was analyzed using KEGG software^1^ to facilitate the related biological interpretation.

## Results

### Protein Expression and mAb Preparation of MCR-1

**FIGURE 1:**
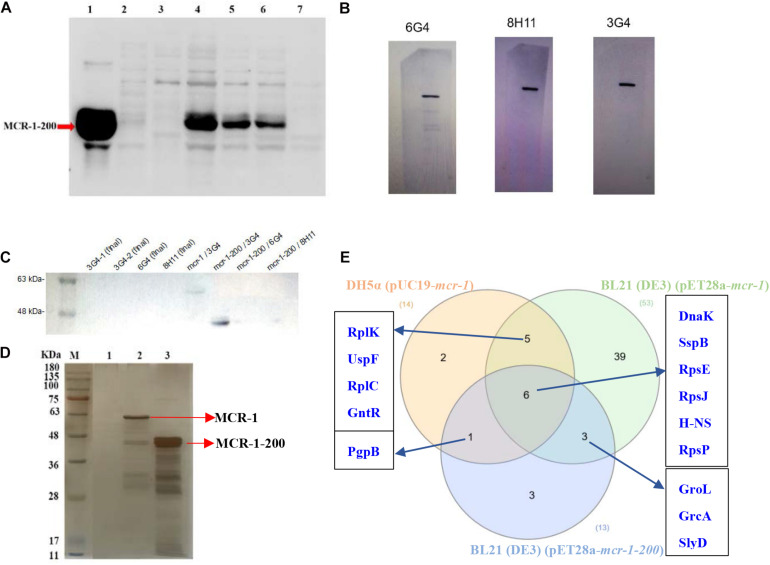
Characterization of the developed Co-IP assays. **(A)** MCR-1 catalytic domain expression in cell lysate. Lane 1–3: purified protein eluted from Ni-NTA column by the elution buffer, wash buffer, and binding buffer, respectively; Lane 4–5: total cellular protein in supernatant and pellets of the sonicated cell lysates, respectively; Lane 6–7: total cellular protein induced expression with IPTG and total cellular protein without induction, respectively. **(B)** Anti-MCR-1 mAbs validation using the cell lysate through regular Western blot method. **(C)** Comparison of the prepared anti-MCR-1 mAbs in Co-IP. **(D)** Representative silver-stained SDS-gel of the eluted binding proteins mixtures using 3G4 mAb bait. M: prestained protein ladder 11–180 KDa; Lanes 1–3: *E. coli* BL21 (DE3) (pET28a), BL21 (DE3) (pET28a-*mcr-1*), and BL21 (DE3) (pET28a-*mcr-1-200*), respectively. **(E)** The Venn diagrams of the identified MCR-1 interacting proteins in three different *E. coli* strains.

Recombinant plasmid pET28a-mcr-1 was induced by IPTG to express a 6 × His-tagged recombinant protein at the N-terminus, consisting of 541 amino acids. The molecular weight of MCR-1 full-length protein after fusion expression was about 66 KDa. The catalytic domain of MCR-1 was induced and expressed by recombinant plasmid pET28a-mcr-1-200. The catalytic domain of MCR-1 consisted of 341 amino acids and its molecular weight was about 46 KDa. IgG positive hybridoma cells belonging to three isotypes-IgG1 (3G4, 6G4, and 8H11) were obtained. The anti-MCR-1 mAbs were purified from ascites fluid by protein G resin affinity chromatography. The cell lysate of the constructed *E. coli* DH5α (pUC19-*mcr-1*) strains was used for anti-MCR-1 mAb validation through regular Western blot method. His6-MCR-1 bands were detected by anti-His6 mAb (Figure 1A) and MCR-1 were successfully recognized by newly prepared anti-MCR-1 mAbs (Figure 1B). Then we compared the suitability of an antibody for Co-IP in a small-scale experiment followed by western blotting. We found that the anti-MCR-1 mAb 3G4 had no cross-linking efficiency and optimal performance in pulling down the target protein (Figure 1C).

### Interactome Profile of MCR-1

In an effort to better understand the mechanistic role of MCR-1, Co-IP, and mass spectrometry were used to identify the interacting proteins of MCR-1 in *E. coli*. Corresponding strong band at the expected size of MCR-1 and MCR-1-200 and a large number of other immunoprecipitated protein bands were shown using SDS-PAGE and silver stained (Figure 1D). We identified a total of 53 interacting proteins linked with ribosomal proteins (RplJ, RplK, RplO, RplL, RplC, RpmC, RplF, RplD, RplI, RpsU, RpsE, RpsJ, RpsP, and RpsN), stress response proteins (DnaK and SspB), metabolism (IDH1, SdhB, AcnB, ATPF0B, and GatB) and drug efflux system (AcrA, Lpp, OmpA, Pal, and TolC) in *E. coli* BL21 (DE3) (pET28a-*mcr-1*) (Table 2). In addition, we identified a total of 14 interacting proteins in *E. coli* DH5α (pUC19-*mcr-1*) and these interacting proteins mainly included ribosomal proteins (RpsE, RplK, RpsJ, RpsP, and RplK), DNA-binding proteins (H-NS, GntR, and EF-Tu) and stress response proteins (DnaK, GroL, and SspB) (Supplementary Table 2). A total of 13 interacting proteins including ribosomal proteins (RpsJ, RpsE, and RpsP), DNA-binding proteins (H-NS, HupA, HupB, and HofN) and stress response proteins (DnaK and SspB) were identified in the soluble domain expression vector *E. coli* BL21 (DE3) (pET28a-*mcr-1-200*) (Supplementary Table 3). The number of MCR-1-interacting proteins identified in full-length protein-expressing strains BL21 (DE3) (pET28a-*mcr-1*) was significantly higher than that in catalytic domain-expressing strains BL21 (DE3) (pET28a-*mcr-1-200*). Next, we analyzed all of the MCR-1-interacting proteins using InteractiVenn software. Of these proteins, six proteins (DnaK, SspB, H-NS, RpsE, RpsJ, and RpsP) were identified in all the three strains (Figure 1E).

**TABLE 2:** MCR-1 protein interactors identified in *E. coli* BL21 (DE3) (pET28a-mcr-1).

No.	Uniprot ID	Protein	Mass (Da)	Gene
1	P0ACF0	DNA-binding protein HU-alpha	9,529	*hupA*
2	P0ACF8	DNA-binding protein H-NS	15,587	*hns*
3	P0A6Y8	Chaperone protein DnaK	69,130	*dnaK*
4	P0A910	Outer membrane protein A	37,292	*ompA*
5	P0A7W1	30S ribosomal protein S5	17,592	*rpsE*
6	P0AFZ3	Stringent starvation protein B	18,251	*sspB*
7	P0A6N2	Elongation factor Tu	43,457	*tufA*
8	P0ACF4	DNA-binding protein HU-beta	9,220	*hunB*
9	P69776	Major outer membrane lipoprotein Lpp	8,375	*lpp*
10	P02930	Outer membrane protein TolC	53,708	*tolC*
11	P0A7R5	30S ribosomal protein S10	11,728	*rpsJ*
12	P0A9K9	FKBP-type peptidyl-prolyl *cis-trans* isomerase SlyD	21,182	*slyD*
13	P0A7K2	50S ribosomal protein L7/L12	12,288	*rplL*
14	P0ADT8	Uncharacterized protein YgiM	23,062	*ygiM*
15	P0C054	Small heat shock protein IbpA	15,764	*ibpA*
16	P0AEH5	Protein ElaB	11,299	*elaB*
17	P0ADP9	Protein YihD	10,323	*yihD*
18	P0AG55	50S ribosomal protein L6	18,949	*rplF*
19	P0AF36	Cell division protein ZapB	9,629	*zapB*
20	P37903	Universal stress protein F	16,064	*uspF*
21	P0A6Y1	Integration host factor subunit beta	10,645	*ihfB*
22	P45523	FKBP-type peptidyl-prolyl *cis-trans* isomerase FkpA	28,864	*fkpA*
23	P0ADS2	Cell division protein ZapA	12,643	*zapA*
24	Q0TLG8	UPF0325 protein YaeH	15,144	*yaeH*
25	P0A7T3	30S ribosomal protein S16	9,185	*rpsP*
26	P69428	Sec-independent protein translocase protein TatA	9,658	*tatA*
27	P0AG30	Transcription termination factor Rho	47,032	*rho*
28	P0AGE0	Single-stranded DNA-binding protein	18,963	*ssb*
29	P02925	Ribose import binding protein RbsB	30,931	*rbsB*
30	P0A7J7	50S ribosomal protein L11	14,923	*rplK*
31	P64596	Uncharacterized protein YraP	20,073	*yraP*
32	Q0TCG0	50S ribosomal protein L15	14,957	*rplO*
33	Q0TCE1	50S ribosomal protein L3	22,230	*rplC*
34	P0A7M6	50S ribosomal protein L29	7,269	*rpmC*
35	P0A7J3	50S ribosomal protein L10	17,757	*rplJ*
36	P0AG59	30S ribosomal protein S14	11,630	*rpsN*
37	P0ABA0	ATP synthase subunit b	17,310	*atpF*
38	Q0TCE2	50S ribosomal protein L4	22,073	*rplD*
39	P0A6 × 7	Integration host factor subunit alpha	11,347	*ihfA*
40	P0AGJ9	Tyrosine–tRNA ligase	47,896	*tyrS*
41	P0A7R1	50S ribosomal protein L9	15,759	*rplI*
42	P0AEZ3	Septum site-determining protein MinD	29,710	*minD*
43	Q0TD41	30S ribosomal protein S21	8,552	*rpsU*
44	P36683	Aconitate hydratase B	94,009	*acnB*
45	P0AEU7	Chaperone protein Skp	17,677	*skp*
46	P0AE06	Multidrug efflux pump subunit AcrA	42,228	*acrA*
47	P0ACP5	HTH-type transcriptional regulator GntR	36,570	*gntR*
48	Q0TB02	Membrane protein insertase YidC	61,557	*yidC*
49	P07014	Succinate dehydrogenase iron-sulfur subunit	27,379	*sdhB*
50	P37188	Galactitol-specific phosphotransferase enzyme IIB component	10,443	*gatB*
51	P0A912	Peptidoglycan-associated lipoprotein	18,869	*pa1*
52	P0A905	Outer membrane lipoprotein SlyB	15,649	*slyB*
53	P08200	Isocitrate dehydrogenase (NADP)	46,070	*icd*
54	A0A0R6L508	MCR-1	60,428	*mcr-1*

**FIGURE 2:**
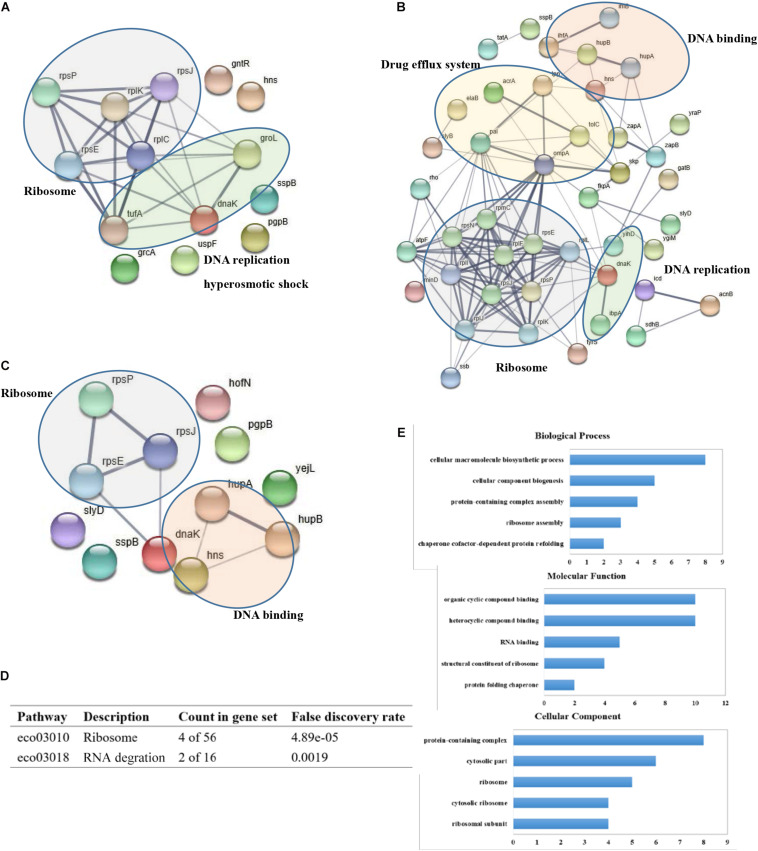
Defining the MCR-1 functional interactome. **(A)** PPI network generated for 14 MCR-1 interactors using experimental evidence in STRING database and medium confidence links (>0.400) in *E. coli* DH5α (pUC19-*mcr-1*). Thickness of edges represent strength of evidence supporting interaction; **(B)** PPI network generated for 53 MCR-1 interactors in *E. coli* BL21 (DE3) (pET28a-*mcr-1*); **(C)** PPI network of the 13 MCR-1 interactors in *E. coli* BL21 (DE3) (pET28a-*mcr-1-200*); **(D)** KEGG pathway analysis of the interacting proteins of MCR-1 in *E. coli* DH5α (pUC19-*mcr-1*); **(E)** GO enrichment analysis of the interacting proteins of MCR-1 in *E. coli* DH5α (pUC19-*mcr-1*).

To elucidate the functional relationships between the MCR-1 interacting proteins, we extracted a PPI network over these proteins from the STRING interaction database. We identified several functional proteins associated with ribosome, DNA replication, and hyperosmotic shock in *E. coli* DH5α (pUC19-*mcr-1*) (Figure 2A). The interacting proteins of MCR-1 in *E. coli* BL21 (DE3) (pET28a-*mcr-1*) were mainly involved in ribosome, drug efflux system, DNA binding, and DNA replication (Figure 2B). And the MCR-1-interacting proteins in *E. coli* BL21 (DE3) (pET28a-*mcr-1-200*) were mainly related with ribosome and DNA binding (Figure 2C). KEGG pathway enrichment analysis of the MCR-1-interacting proteins in *E. coli* DH5α (pUC19-*mcr-1*) found that these proteins were mainly involved in ribosome and RNA degradation (Figure 2D). And the KEGG pathway of the MCR-1-interacting proteins identified in *E. coli* BL21 (DE3) (pET28a-*mcr-1*) were referred to ribosome, RNA degradation, and cationic antimicrobial peptide (CAMP) resistance (Supplementary Figure 1A). Interestingly, multidrug efflux pump AcrA and TolC was identified important interacting partners of MCR-1 (Table 2). Outer membrane lipoprotein SlyB contributes to membrane integrity. YidC is required for the insertion and/or proper folding of integral membrane proteins into the membrane. The discovery of these interacting proteins demonstrated that MCR-1 might affect the expression and regulation of membrane proteins to cause drug efflux during the PEA modification of bacterial cell membrane lipid A.

To identify putative functional processes associated with MCR-1-interacting proteins, we performed GO analysis. In *E. coli* DH5α (pUC19-*mcr-1*), the top-ranked categories of Biological Process analysis were cellular macromolecule biosynthetic process, cellular component biogenesis, protein-containing complex assembly (Figure 2E), suggesting that MCR-1 is related to protein biosynthetic process. In addition, Molecular Function of the MCR-1 interacting partners referred to organic cyclic compound binding, heterocyclic compound binding and RNA binding (Figure 2E). It indicates that MCR-1 may be involved in nucleic acid binding. Cellular Component analysis showed that these proteins were related to protein-containing complex, cytosolic part, and ribosome (Figure 2E), which implies that MCR-1 is likely to participate in protein biosynthesis. GO analysis of MCR-1-interactiong proteins in *E. coli* BL21 (DE3) (pET28a-*mcr-1*) were shown in Supplementary Figures 1B–D.

### Interaction Analysis of SspB With MCR-1

**FIGURE 3:**
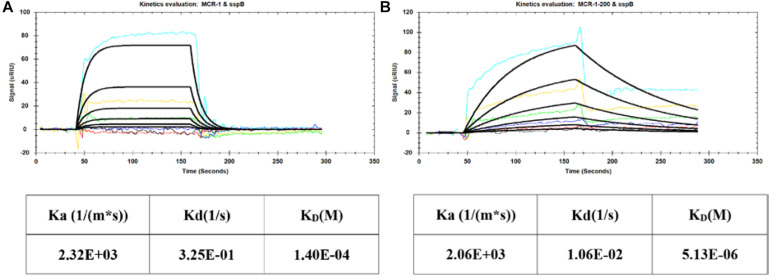
SPR interaction analysis of MCR-1 and SspB protein. **(A)** Kinetic evaluation of MCR-1 full length protein and SspB; **(B)** Kinetic evaluation of MCR-1-200 catalytic domain protein and SspB. The SspB was diluted with PBST running buffer in the concentration range of 0.156–5 μM with twofold dilution series.

To confirm the reliability of the developed Co-IP method and the effects of the identified MCR-1-interacting proteins, we investigated the binding ability of the target protein MCR-1, MCR-1-200, and SspB on the sensor chip. The SPR responses were obtained when the protein solution (0.156–5 μM, twofold dilution) was injected onto the sensor chip. The K_*D*_ values for the association between the MCR-1 full length protein and the SspB were 1.40 × 10^–4^ and 5.13 × 10^–6^ M for catalytic domain protein MCR-1-200, respectively (Figures 3A,B). These results demonstrated that MCR-1 was directly interacted with SspB in colistin-resistant *E. coli* strains.

## Discussion

With the rapid spread of the *mcr-1*, its public health problems and human health threats need to be solved urgently. The identification of functional interaction partners is fundamental to understanding the role of proteins and the mechanism of action *in vivo*. This is particularly challenging because bacteria will continue to produce adaptive changes under the pressure of antibiotics. Using a reliable Co-IP assay couple with mass spectrometry, we reported here the first functional interactome of MCR-1 and identified the key interacting proteins in colistin-resistant *E. coli* strains.

Our data indicate that MCR-1 interacts with multiple types of proteins. Among the interacting proteins identified by mass spectrometry, the most enriched groups of proteins associated with MCR-1 were the components of the ribosome and cellular stress response (Figure 2D). We speculate that MCR-1 interact with ribosome-associated proteins to affect protein biosynthesis in conferring resistance by modifying the colistin target, catalyzing transfer of phosphoethanolamine (PEA) onto the glucosamine saccharide of lipid A in the bacterial outer membrane. Our previous research indicated that the expression of some ribosomal proteins was disturbed in the construct *E. coli* strains carrying *mcr-1* and the bacteria could enhance protein synthesis in order to adapt to drug selection pressure (Li et al., 2019). Evaluating the likelihood and functional impact of the ribosome-associated interacting partners of MCR-1 will be an interesting follow up of this study.

As known, AcrAB-TolC multidrug resistance pump provides the Gram-negative bacteria with the necessary means to adapt drug pressure (Nikaido and Takatsuka, 2009). AcrA is the adapter component that associates the inner membrane pump with the TolC outer membrane channel (Fralick, 1996; Symmons et al., 2009). Our previous research found that MCR-1 not only caused the PEA modification of bacterial cell membrane lipid A, but also affected the efflux of polymyxin through disturbing the expression of efflux pump proteins involved in CAMP resistance pathway (Li et al., 2019). This study further confirmed that AcrA and TolC were important interacting membrane proteins of MCR-1 referred to drug efflux process. This finding indicated that MCR-1 might cause bacterial cell membrane to undergo the PEA molecule modification, which might also cause efflux pumps to participate in this biological process. The identification of the interacting partners SlyB and YidC showed that the importance of membrane protein integrity in *mcr-1*-mediated colistin resistance in *E. coli*. Lpp was a protein required for maintaining structural and functional integrity of bacterial cell envelope (Stenberg et al., 2005). Lpp was an integral component of cell outer membrane and seemed to interact with TolB, Pal, and TonB. We also found that H-NS interacted with DnaK, Lpp, HupA, and HupB in our study (Figure 2B). The regulatory protein H-NS controlled the lipid A palmitoylation mediated by the PagP enzyme (Chalabaev et al., 2014). Future work is needed to uncover the role of the promiscuous interaction of MCR-1 with two-component efflux pump AcrA-TolC and the contribution of other membrane proteins of the MCR-1 interactome during colistin resistance.

The stringent response is a general bacterial stress response that allows bacteria to adapt and survive adverse conditions. The cellular response to stress is orchestrated by the expression of a family of proteins termed heat shock proteins (e.g., DnaK) that are involved in the stabilization of basic cellular processes to preserve cell viability and homeostasis. Here, we found DnaK was a chaperone protein that interacted with MCR-1 (Figure 1E and Table 2). Chaperone protein DnaK was a multifunctional chaperone of highly conserved HSP70 family which assisted in protein folding, disaggregation, and remodeling of protein complexes (Calloni et al., 2012; Zahn et al., 2013). DnaK functions as a central hub to interact with a large number of proteins to regulate ribosomal biogenesis in *E. coli* (Zhang et al., 2016). DnaK has been proven to play an important role in the stress resistance of microorganism and may associate with the fitness cost reduction for *mcr-1*-carrying plasmids (Genevaux et al., 2007; Ma et al., 2018). We elucidate that DnaK might assist in the regulation of ribosomal biogenesis and affect the lipopolysaccharide modification. SspB, another protein that has been shown to interact with MCR-1 using Co-IP and SPR (Figure 3). Stringent starvation protein B (SspB) enhanced recognition of SsrA-tagged proteins by the ClpX-ClpP protease and regulated the protein expression during exponential and stationary-phase growth (Levchenko et al., 2000). H-NS was a global DNA-binding transcriptional dual regulator and implicated in transcriptional repression. RpsE, RpsJ, and RpsP are components of 30S ribosomal subunit and play an important role in ribosome biosynthesis. RpsE linked to the functional center of the 30S ribosomal subunit and was implicated in translational accuracy (Wimberly et al., 2000; Kirthi et al., 2006). RpsJ is involved in the regulation of ribosomal RNA biosynthesis by transcriptional antitermination (Luo et al., 2008; Weisberg, 2008). RpsP is essential for the viability of *E. coli* and plays an important role in the assembly of the 30S ribosomal subunits (Persson et al., 1995). So we predicted that MCR-1 interacted with H-NS to inhibit the DNA transcription and linked with ribosome proteins (RpsE, RpsJ, RpsP, etc.). Importantly, the identification and validation of MCR-1 interaction partner SspB with proven relevance demonstrate the power of our new Co-IP assay and provide reliable protein targets to advance our understanding of the *mcr-1*-mediated colistin resistance.

In conclusion, we define the functional interactome profile of colistin resistant protein MCR-1 in *E. coli* strains using Co-IP and mass spectrometry. Our study has uncovered a conceivable mechanism that MCR-1 influences the protein biosynthesis through the interaction with ribosomal protein. Multidrug efflux pump AcrA and TolC involved in the cationic antimicrobial peptide (CAMP) resistance pathway were identified as important interacting partners of MCR-1. Our data illustrates the interacting network of MCR-1 in colistin resistance and can provide valuable information to accurately understand its function and the mechanism of action at a deeper level.

## Data Availability Statement

The datasets presented in this study can be found in online repositories. The names of the repository/repositories and accession number(s) can be found in the article/Supplementary Material.

## Ethics Statement

The animal study was reviewed and approved by Chinese laws and guidelines that were approved by the Animal Ethics Committee of China Agricultural University.

## Author Contributions

BS, JS, YW, and XX conceived and designed the experiments. HL, YW, and QC performed the experiments. HL, YW, QC, and XX analyzed the data. HL, YW, and BS wrote the manuscript. All authors contributed to the article and approved the submitted version.

## Conflict of Interest

The authors declare that the research was conducted in the absence of any commercial or financial relationships that could be construed as a potential conflict of interest.

## Footnotes

1 http://www.genome.jp/kegg/
