# Supplementary material for: Identification of Functional Interactome of Colistin Resistance Protein MCR-1 in Escherichia coli
Source: Front Microbiol. 2021 Jan 25;11:583185. doi: 10.3389/fmicb.2020.583185 (PMC7868338; doi:10.3389/fmicb.2020.583185)

Supplementary Material

# Table S1 Primers used in this study.

| **Primer** | **Sequence (5’-3’)** |
| --- | --- |
| pET28a-mcr-1_F | CATGCCATGGTGCACCACCACCACCACCACATGATGCAGCATACTTCTGTGTGGTACCGACG |
| pET28a-mcr-1_R | CAAAGACCGCACCGCATTCATCCGCCTCGAGCGG |
| pET28a_mcr-1-200-F | CATGCCATGGTGCACCACCACCACCACCACTCGGTGGGTAAGCTTGCCAGTATTG |
| pET28a_mcr-1-200-R | CCGCTCGAGTCAGCGGATGAATGCGGTGC |

**Table S2 MCR-1 protein interactors identified in *E. coli* DH5α(pUC19-*mcr-1*).**

| **No.** | **Uniprot ID** | **Protein** | **Mass (Da)** | **Gene** |
| --- | --- | --- | --- | --- |
| 1 | P0A6Y8 | Chaperone protein DnaK | 69130 | *dnaK* |
| 2 | P0AFZ3 | Stringent starvation protein B | 18251 | *sspB* |
| 3 | P0A7W1 | 30S ribosomal protein S5 | 17592 | *rpsE* |
| 4 | P0A6N2 | Elongation factor Tu | 43457 | *tufA* |
| 5 | P0ACF8 | DNA-binding protein H-NS | 15587 | *hns* |
| 6 | P0A7J7 | 50S ribosomal protein L11 | 14923 | *rplK* |
| 7 | P0A7R5 | 30S ribosomal protein S10 | 11728 | *rpsJ* |
| 8 | P0A6F5 | 60 kDa chaperonin | 57464 | *groL* |
| 9 | P0A7T3 | 30S ribosomal protein S16 | 9185 | *rpsP* |
| 10 | Q0TEQ9 | Autonomous glycyl radical cofactor | 14316 | *grcA* |
| 11 | P37903 | Universal stress protein F | 16064 | *uspF* |
| 12 | Q0TCE1 | 50S ribosomal protein L3 | 22230 | *rplC* |
| 13 | P0A924 | Phosphatidylglycerophosphatase B | 29117 | *pgpB* |
| 14 | P0ACP5 | HTH-type transcriptional regulator GntR | 36570 | *gntR* |
| 15 | A0A0R6L508 | MCR-1 | 60428 | *mcr-1* |

**Table S3 MCR-1-interacting proteins identified in *E. coli* BL21(DE3)(pET28a-*mcr-1-200*).**

| **No.** | **Uniprot ID** | **Protein** | **Mass (Da)** | **Gene** |
| --- | --- | --- | --- | --- |
| 1 | P0A6Y8 | Chaperone protein DnaK | 69130 | *dnaK* |
| 2 | P0AFZ3 | Stringent starvation protein B | 18251 | *sspB* |
| 3 | P0A7R5 | 30S ribosomal protein S10 | 11728 | *rpsJ* |
| 4 | P0A7W1 | 30S ribosomal protein S5 | 17592 | *rpsE* |
| 5 | P0ACF8 | DNA-binding protein H-NS | 15587 | *hns* |
| 6 | P0A9K9 | FKBP-type peptidyl-prolyl cis-trans isomerase SlyD | 21182 | *slyD* |
| 7 | P0ACF0 | DNA-binding protein HU-alpha | 9529 | *hupA* |
| 8 | P0ACF4 | DNA-binding protein HU-beta | 9220 | *hupB* |
| 9 | P64634 | Putative DNA utilization protein HofN | 20783 | *hofN* |
| 10 | P0A7T3 | 30S ribosomal protein S16 | 9185 | *rpsP* |
| 11 | P0AD24 | UPF0352 protein YejL | 8283 | *yejL* |
| 12 | P0A924 | Phosphatidylglycerophosphatase B | 29117 | *pgpB* |
| 13 | Q00191 | Protein TraI | 81742 | *traI* |
| 14 | A0A0R6L508 | MCR-1 | 60428 | *mcr-1* |

**Figure S1.** Kegg pathway and GO enrichment analysis of MCR-1-interacting proteins in *E. coli* BL21(DE3)(pET28a-*mcr-1*). (A) KEGG pathway analysis of the interacting proteins of MCR-1 categorized into functional groups; (B-D) GO analysis of the interacting proteins of MCR-1.


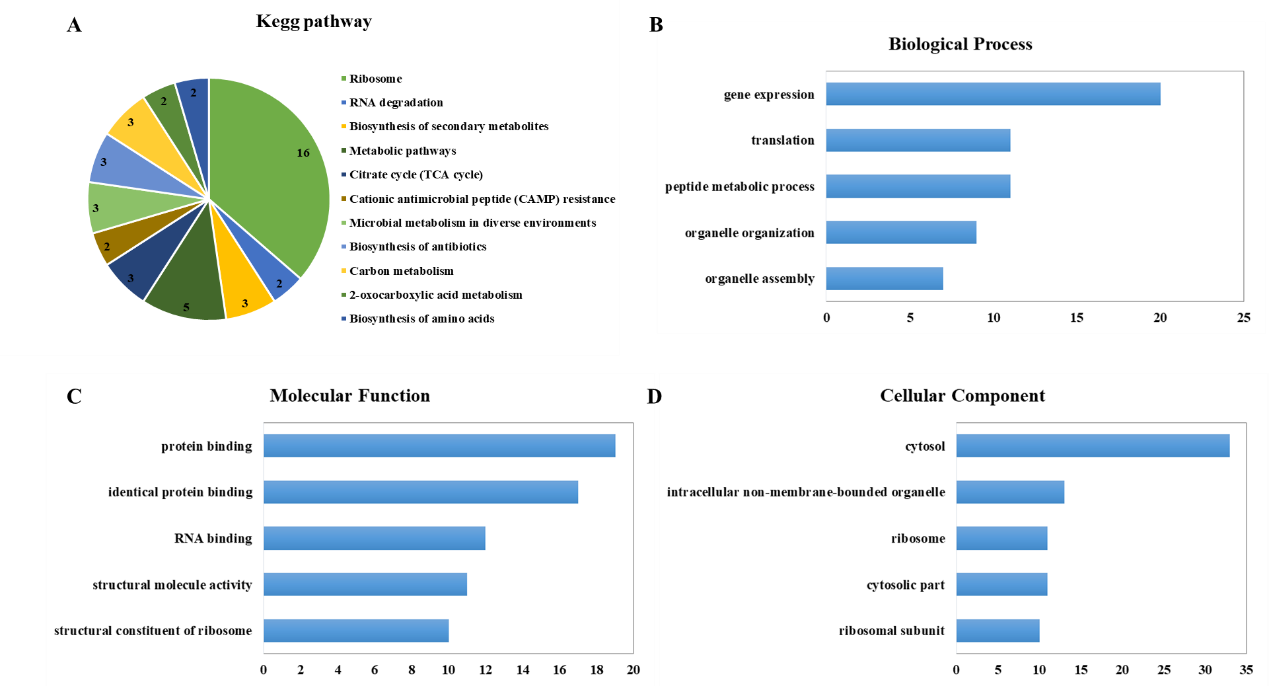

Supplement: Supplementary file 1 [file Data_Sheet_1.docx]
